# Curcumin and zinc co-supplementation along with a loss-weight diet can improve lipid profiles in subjects with prediabetes: a multi-arm, parallel-group, randomized, double-blind placebo-controlled phase 2 clinical trial

**DOI:** 10.1186/s13098-022-00792-2

**Published:** 2022-01-28

**Authors:** Majid Karandish, Hassan Mozaffari-khosravi, Seyed Mohammad Mohammadi, Bahman Cheraghian, Maryam Azhdari

**Affiliations:** 1 Nutrition and Metabolic Diseases Research Center, Clinical Sciences Research Institute, Ahvaz Jundishapur University of Medical Sciences, Ahvaz, Iran; 2 Department of Nutrition, School of Public Health, Shahid Sadoughi University of Medical Sciences and Health Services, Yazd, Iran; 3 Associate Professor of Endocrinology & Metabolism, School of Medicine, Shahid Sadoughi University of Medical Sciences and Health Services, Yazd, Iran; 4 Alimentary Tract Research Center, Clinical Sciences Research Institute, Department of Biostatistics and Epidemiology, School of Health Sciences, Ahvaz Jundishapur University of Medical Sciences, Ahvaz, Iran

**Keywords:** Curcumin, Zinc, Dietary intake, Liver enzymes, Lipid profiles, Prediabetes

## Abstract

**Background:**

Diabetes is one of the major public health concerns. Prediabetes can increase the risk of developing some non-communicable diseases such as type 2 diabetes. Given the increasing trend of prediabetes, it is critical to control it and prevent its complications. Curcumin is a major bioactive component of turmeric. Zinc is an antioxidant nutrient. The present trial aimed to evaluate the effect of curcumin and zinc co-supplementation along with a loss-weight diet on serum lipid profiles in overweight or obese patients with prediabetes.

**Methods:**

Eighty-four participants were randomized to four groups (curcumin (500 mg/day), zinc (30 mg/day), “curcumin and zinc”, and placebo) for 90 days. Serum total cholesterol (TC), low-density lipoprotein (LDL), high-density lipoprotein (HDL), triglycerides (TG), non-HDL, HDL/LDL ratio, weight, BMI, waist circumstance (WC), hip circumstance (HC), physical activity (PA) and dietary intake were determined pre and post-intervention. This study will be conducted at Yazd Diabetes Research Clinic, Shahid Sadoughi University of Medical Sciences.

**Results:**

Totally, 82 participants were included in the final analysis. After the adjusted PA effect, changes in serum TG (adjusted p = 0.001), LDL (adjusted p = 0.035), non-HDL (adjusted p = 0.003), HDL/LDL ratio (adjusted p = 0.002), and HDL (adjusted p < 0.0001) revealed a significant difference between the groups. However, the changes in weight (adjusted p = 0.004) and BMI (adjusted p = 0.006) were significant but the changes in dietary intake, PA, WC, and HC were non-significant (adjusted p ≥ 0.05). Despite that there was a significant difference for post-intervention HDL levels (adjusted p = 0.016), other lipid profiles showed no significant difference (adjusted p ≥ 0.05).

**Conclusion:**

The beneficial effects of “curcumin and zinc” co-supplementation was reported for the changes of some lipid profiles (TG, LDL, HDL, non-HDL, and HDL to LDL ratio), BMI, and weight with no positive effects on TC, dietary intake, PA, WC, and HC. Therefore, it may play a potential role in the prevention of macro and microvascular complications.

*Trial registration* The project is a registered clinical trial (Registration number: IRCT20190902044671N1, Iranian Registry of Clinical Trials (IRCT), registered October 11, 2019.

**Supplementary Information:**

The online version contains supplementary material available at 10.1186/s13098-022-00792-2.

## Introduction

Prediabetes status is defined as impaired glucose tolerance (IGT), impaired fasting glucose (IFG), and/or HbA_1_C 5.7–6.4% [1]. Some complications of prediabetes include macrovascular (cardiovascular disease (CVD), stroke, peripheral artery disease), microvascular (retinopathy, neuropathy, and nephropathy), and type 2 diabetes mellitus (T2DM) [1, 2]. The prevalence of prediabetes among some populations was related to abdominal or visceral obesity, dyslipidemia with high triglycerides (TG), high low-density lipoprotein cholesterol (LDL-C), high total cholesterol (TC), low high‐density lipoprotein-cholesterol (HDL-C), and/or hypertension [1, 3, 4]. Moreover, the high prevalence of obesity, dyslipidemia, and T2DM along with cardiovascular complications was reported in the past decade. The physiological, metabolic, or/and biochemical characteristics are abnormal in prediabetic patients. Since the adverse changes in both lipid and glucose concentrations were reported in prediabetes and T2DM, the target of the novel therapeutic approaches is the simultaneous improvement of glucose and lipid control. Therefore, early diagnosis and effective intervention are critical to control prediabetes and delay its complications [5]. Physical activity (PA) and dietary modifications can consider as the key lifestyle interventions. The pharmacological interventions usually apply in prediabetic patients without a positive response to lifestyle interventions [2]. Recent researches have shown too much attention to phytochemicals and the antioxidant trace elements to treat and control some diseases (T2DM, prediabetes, metabolic syndrome, CVD, and chronic venous insufficiency (CVI)) [6–11].

Curcumin (diferuloylmethane) is a bioactive constituent of Curcuma longa L. (Zingiberaceae), commonly known as turmeric with diverse pharmacological and biological properties [7]. Curcumin has shown beneficial effects on glycemic parameters (fasting plasma glucose (FPG), 2-h postprandial (2hpp), HbA_1_C, insulin, insulin sensitivity (IS) and insulin resistance (IR)) in prediabetic patients [11], lipid profiles (TG, TC, LDL-C, and HDL-C) and blood pressure (BP) in T2DM [8] and metabolic syndrome [6]. The finding of a meta-analysis of randomized controlled trials showed curcumin may play a protective role in patients at risk of CVD by improving serum lipid levels [10].

Zinc is a trace element that has health benefits in the various aspects of metabolism through antioxidant and anti-inflammatory properties, and cofactor of many enzymes [12–14]. Zinc apart from its role in improving glycemic markers in patients with prediabetes [11] and T2DM [15], plays a notable role in lipid metabolism [16] that can control CVD, one of the current complications in prediabetes and diabetes. However, the findings of some trials or meta-analyses of trials indicated that zinc supplementation improves some lipid profiles, some studies did not show it [17–19].

On the one hand, the results of the effect of curcumin or zinc were inconsistent in previous studies, and on the other hand, there was no trial with aim of evaluating the effect of curcumin and zinc co-supplementation on lipid profiles in prediabetic patients. The present multi-arm, parallel-group, randomized, double-blind placebo-controlled phase 2 clinical trial aimed to evaluate the effect of curcumin and zinc co-supplementation along with loss-weight diet on serum lipid profiles (TG, LDL, HDL, TC, non-high-density lipoprotein cholesterol (non-HDL), and HDL to LDL ratio) in overweight or obese patients with prediabetes.

## Methods

The details of the method and materials were previously published [20].

### Participants and sampling

All 84 eligible prediabetic adults with written informed consent were recruited from Yazd Diabetes Research Clinic and enrolled in the present randomized, clinical trial (RCT) for 90 days. The inclusion criteria were included women or men (18–50 years old for men and 18 years- before menopause for women) with prediabetes according to the ADA guidelines [1] and 25 < BMI > 35. Patients were excluded from the trial if a diagnosis of any types of malignancies/cancers, cardiovascular, kidney, lung, endocrine, autoimmune, inflammatory, and neurological diseases, and/ or hypertension, taking BP, glucose or lipid-lowering drugs; taking multivitamin-mineral supplements for three months before or during the intervention; a history of weight loss surgery in the last year, a weight-loss plan in the last 3 months; receiving from a weight-loss medicine or program; lactating, pregnant or planning to get pregnant; unwillingness/the compliance of less than 80% during the intervention, or no signed informed consent.

Considering that the present study was a part of comprehensive research, the sample size was measured for all variables and the largest sample size was considered. The sample size was calculated using both WinPepi statistical program (Version 11.4: Abramson, 2011) and a parallel design randomized controlled trial formula (formula was previously published) [20]. Twenty-one participants in each group were required (80% power at 0.05 significance level and accounting for a drop-out rate of 10%) [20].

### Study setting and design

This multi-arm, parallel-group, randomized, double-blind, placebo-controlled, phase 2 clinical trial was reported according to the CONSORT statement (Consolidated Standards for Reporting Trials). The method of block randomization with a block size of 4 using a computer-generated random number sequence and the allocation concealment (assigning the unique codes to the eligible participant) by a blinded independent statistician and the pharmacist (who was not involved during the study), respectively. All the assessments in the trial were made by the investigators blinded to the treatment allocation. The participants were not informed about the type of supplement. The supplements were delivered to the participants according to the allocation on the 1st, 30th, and 60th day.

The eligible participants were randomized into four parallel groups, (1) the curcumin group: curcumin supplement (500 mg-BCM95/ Curcugreen capsule, M/s Arjuna Natural Pvt Ltd., India) and placebo for zinc (lactose); (2) the zinc group: zinc supplement (30 mg zinc in form of zinc gluconate tablet, Dineh company, Iran) and the placebo for curcumin (roasted rice powder); (3) the “curcumin & zinc” group: both a curcumin capsule and a zinc tablet; (4) the placebo group: both a placebo capsule for curcumin and a placebo tablet for zinc. Dosages were selected based on the previous studies [21, 22]. The placebo was identical in texture, weight, and appearance to its active supplement. A curcumin capsule (supplement or placebo) and a zinc tablet (supplement or placebo) were prescripts once daily after and before breakfast for 90 days, respectively. All participants have followed a standard, individualized hypo-caloric diet designed with at least a 7% weight loss including 45–55%, 25–35%, and 10–20% of their calories from carbohydrate, fat, and protein, respectively, and PA for 150 min per week for improving lifestyle. The nutritional modifications, including adherence to the diet and avoiding excessive intake of high-fat and/or sugar products, were recommended by a nutritionist.

This trial was approved by the Medical Ethics Committee of Ahvaz Jundishapur University of Medical Sciences, Ahvaz, Iran; (Ethical code: IR.AJUMS.REC.1398.504) and it is prospectively registered in the Iranian Registry of Clinical Trials (Registration number: IRCT20190902044671N1).

### Follow-up

In order to control the participants for taking supplements and placebo, they were followed up using messaging tools or the telephone, daily or weekly. The lifestyle modifications were individually recommended for all participants who were visited at the end of 30-day periods. Moreover, pill counts were recorded every 30 days to assess adherence to the supplement. All adverse events were documented and reported to the Data Monitoring Committee and the Ethics Committee of the Ahvaz Jundishapur University of Medical Sciences.

### Data collection and outcome measures

The general demographic and medical history data (age, gender, education levels, duration of prediabetes, and family history of prediabetes or hypertension) were obtained from each participant by interview. The measurement of weight and height were recorded to assess body mass index (BMI) (kg/m^2^) which was calculated by this equation: weight (kg)/height (m^2^). The waist and hip circumstances were assessed by a non-elastic and flexible tape.

The short form of the International Physical Activity Questionnaire (IPAQ-SF) was used for the evaluation of PA. PA was reported as metabolic equivalents (METs)-minutes per week (MET-min/week) and categorized into three groups including active (PA > 3000 MET-min/week), moderate (PA = 600–3000 MET-min/week), and inactive (PA < 600 MET-min/week) [23].

Diastolic Blood Pressure (DBP) and Systolic Blood Pressure (SBP) were measured at the baseline of the study according to the American Heart Association protocol (blood pressure was assessed in a quiet place after 5 min of rest in sitting status without crossed legs and unsupported back and arms) [24]. The side effects of supplements were recorded during the study and also liver enzymes were evaluated to monitor possible side effects of supplements.

Blood samples were collected in an EDTA tube and centrifuged to obtain serum. Serum TG, HDL, TC, FPG (mg/dL) levels, HbA1C (%), alanine transaminase (ALT), and aspartate transaminase (AST) were measured immediately after sampling. The values of non-HDL, HDL/LDL ratio, and LDL-C were calculated by the following the formula: TC minus HDL-C, HDL (mg/dL) divided by LDL (mg/dL), and Friedewald formula (TC (mg/dL) − HDL-c (mg/dL) − TG (mg/dL)/5), respectively [25].

The outcomes included serum lipid profiles and anthropometric measurements which were measured at the baseline and day 90 after the intervention.

## Statistical analysis

All analyses were performed using a statistical software package (SPSS), version 22.0 (SPSS, Inc, Chicago, Illinois, USA). Statistical significance was determined at p < 0.05. Normal distribution and the homogeneity of variances for quantitative variables were checked by Kolmogorov–Smirnov test and Leven’s tests, respectively. Data were reported as mean ± standard deviation (SD) and median (interquartile range) for normally and non-normally distributed data, respectively. Within-group comparisons were performed using a paired-samples t-test. Between-group comparisons for variables were carried out using a one-way ANOVA with a post hoc (LSD) analysis test and Kruskal–Wallis for normally and non-normally distributed data, respectively. The changes (pre-post intervention) were calculated based on the difference of variables from the baseline (pre-intervention) to the end (post-intervention) of the study. However, there was no significant difference for pre-intervention, post-intervention, [11] and changes of dietary intake (Additional file 1: Table A2), a significant difference was previously reported in PA levels at the baseline and changes in BMI and weight. Therefore, the adjustment was applied using univariate analysis of covariance (ANCOVA) and a general linear model to control the effects of the baseline PA or/and changes of BMI and weight from the baseline.

## Results

**Fig. 1:**
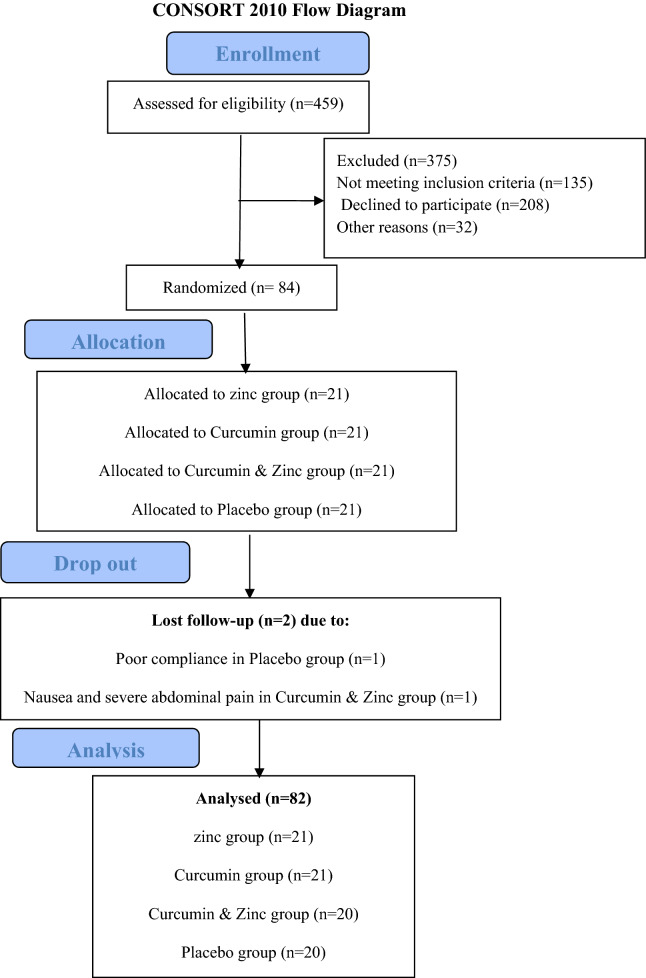
Consolidated Standards of Reporting Trials (CONSORT) flow chart– trial protocol

A total of 82 participants completed the 90 days of the trial. Two patients lost the follow-up due to poor compliance and nausea and severe abdominal pain in the “curcumin and zinc” and placebo group (1 patient in each group), respectively. Figure 1 showed the flow diagram of the study. The mild side effects including headache (n = 2) in curcumin group, headache (n = 1) in zinc group, headache (n = 1), nausea (n = 2), dizziness (n = 1) in “curcumin and zinc” group, and headache (n = 1), nausea (n = 1), hearing impairment (n = 1) in placebo group were reported by the participants.

The mean duration of prediabetes (months) and age (year) of participants was reported 2.31 ± 1.37 and 36.04 ± 6.61, respectively. As set out in Table 1, the general and clinical characteristics of the participants were not found any significant difference between the four groups at the baseline except PA levels (p = 0.03). Moreover, there was no significant difference in pre-intervention biochemical measurements of the participants between groups (Additional file 1: Table A1).

**Table 1:** General and Clinical characteristics of the participants at the baseline

Variables	Groups				P-value
	Placebo N = 20	Curcumin N = 21	Zinc N = 21	Curcumin and Zinc N = 20	
Qualitative variables					
Age (year)	34.19 ± 7.03	36.95 ± 7.23	38.19 ± 4.87	34.48 ± 6.45	0.14
Weight (kg)	84.67 ± 9.78	82.6 ± 8.03	81.81 ± 13.08	80.57 ± 10.17	0.65*
Body mass index (kg/m^2^)	30.97 ± 2.33	30.46 ± 2.75	29.5 ± 2.82	29.95 ± 2.56	0.32*
Waist circumstance (cm)	108.01 ± 10.18	107.44 ± 9.9	103.39 ± 11.75	106.77 ± 10.19	0.493*
Hip circumstance (cm)	111.76 ± 7.16	112.47 ± 6.84	109.79 ± 6.04	108.88 ± 5.15	0.242*
Physical activity	445 (396–853.88)	990 (495–1860)	396 (396–1440)	396 (396–982)	**0.03**^**#**^
Systolic blood pressure (mmHg)	110.56 ± 9	117.7 ± 6.9	115.7 ± 7.8	114.5 ± 8.2	0.437
Diastolic blood pressure (mmHg)	70.43 ± 4.3	75 ± 3.6	73 ± 5.1	73.3 ± 4.2	0.644
Fat (gr)	30.47 ± 3.85	32.03 ± 3.42	32.03 ± 3.6	30.25 ± 4.1	0.26*
Quantitative variables					
Gender (female)	12 (60)	16 (76.2)	15 (71.5)	13 (65)	0.837^#^
Education levels					
University education	10 (50)	14 (66.7)	12 (57.1)	12 (60)	0.585^#^
Diploma	6 (30)	6 (28.6)	4 (19.1)	6 (30)	
Under diploma	4 (20)	1 (4.8)	5 (23.8)	2 (10)	

Data are presented as mean ± standard deviation (SD) for quantitative variables and frequency (%) for qualitative variables

^*^P-value was calculated for the comparison variables between four groups using one-way analysis of variance (one-way ANOVA)

^#^P-value was calculated for the comparison variables between four groups using Kruskal–Wallis

P-value < 0.05 was considered significant

For data with both adjusted and crude analyses, only adjusted analyses were reported. Please refer to tables for crude analysis. No significant difference was shown for changes (pre-post) in dietary intake (energy, protein, carbohydrate, and fat) (adjusted p ≤ 0.05) (Additional file 1: Table A2).

The comparison of changes in liver enzymes, anthropometry measurements, and physical activity was depicted in Table 2. The changes in AST, ALT, WC, HC, and PA were non-significant after adjusting the baseline PA effect (adjusted p ≥ 0.05). After removing PA effects, the changes in weight and BMI were significant (adjusted p = 0.004 and 0.006, respectively). In comparison to the placebo, all three groups receiving the supplement showed significant differences for the changes in weight and BMI after adjusting PA effects (Additional file 1: Table A3).

**Table 2:** Comparison of changes ▀ in liver enzymes, anthropometry measurements, and physical activity between the groups

Variables	Groups				p	Adjusted P*
	Placebo N = 20	Curcumin N = 21	Zinc N = 21	Zinc and curcumin N = 20		
Alanine transaminase (IU/L)	− 0.7 ± 2.96	− 1.05 ± 3.93	− 1.19 ± 3.17	− 1.1 ± 3.23	0.968	0.918
Aspartate transaminase (IU/L)	0.05 ± 2.48	− 0.8 ± 2.48	− 0.09 ± 2.39	0 ± 3.31	0.708	0.426
Weight (kg)	− 2.59 ± 2.45	− 4.88 ± 3.14^b^	− 4.88 ± 2.63^b^	− 5.79 ± 2.77^c^	**0.004**	**0.004**
Body mass index (kg/m^2^)	− 0.92 ± 0.85	− 1.96 ± 1.299^b^	− 1.85 ± 1.01^b^	− 2.09 ± 1.17^c^	**0.004**	**0.006**
Waist circumstance (cm)	− 1.86 ± 2.12	− 3.84 ± 2.86	− 2.95 ± 3.35	− 3.16 ± 2.55	0.154	0.26
Hip circumstance (cm)	− 2.345 ± 2.01	− 4.83 ± 4.16	− 4.08 ± 4.08	− 4.94 ± 3.37	0.081	0.161
Physical activity^#^ (MET·min·wk^−1^)	22.75 (− 74.25, 346)	49.5 (− 738.75, 426)	− 213 (− 213, 346.5)	198 (− 178.5, 532)	0.763	0.98

MET·min·wk^−^1: metabolic equivalent of task minute per week

Data were presented as mean ± standard deviation or median (interquartile (IQR)) for normally and non-normally distributed data, respectively

▀The changes (pre-post intervention) calculated based on the difference of variables from the baseline (pre- intervention) to the end (post intervention) of the study

P was calculated for the comparison variables between four groups using one-way analysis of variance (one-way ANOVA) with post hoc (LSD) analysis

^#^P was calculated for the comparison variables between four groups using Kruskal–Wallis

^*^Adjusted P-value was calculated using ANCOVA or nonparametric ANCOVA; Adjusted for physical activity levels at the baseline

Significant changes with placebo group indicated by ^a^p < 0.05, ^b^p < 0.01, ^c^p < 0.001

The comparison of lipid profiles within and between groups was depicted in Table 3. Moreover, the results of the pairwise comparisons of serum lipid profiles for ANOVA and ANCOVA were shown the Additional file 1: Table A3.

**Table 3:** Comparisons of lipid profiles of the participants between and within the groups

Variables	Groups				p-value	Adjusted p-value*
	Placebo (N = 20)	Curcumin (N = 21)	Zinc (N = 21)	Zinc and curcumin (N = 20)		
Triglyceride (mg/dl)						
Pre-intervention	132.55 ± 25.96	131.48 ± 26.16	125.76 ± 25.74	126.1 ± 27.64	0.776	–
Post-intervention	123.1 ± 18.6	107.48 ± 11.9	114.52 ± 16.93	111.6 ± 18.01	**0.026**	0.286
P-value^▀^	**0.001**	** < 0.001**	**0.001**	** < 0.001**	–	–
Change ▓	− 9.45 ± 10.51	− 24.0 ± 18.97 ^b^	− 11.24 ± 13.821	− 14.50 ± 12.094	**0.007**	**0.001**
Total cholesterol (mg/dl)						
Pre-intervention	186.5 ± 24.91	188.95 ± 23.5	182.52 ± 26.11	181.35 ± 19.98	0.716	–
Post-intervention	184.25 ± 24.4	175.71 ± 17.01	172.67 ± 21.34	170.95 ± 13.45	0.146	0.583
P-value^▀^	0.103	** < 0.001**	**0.001**	** < 0.001**	–	–
Change	− 2.25 ± 5.87	− 13.24 ± 11.52	− 9.86 ± 10.94	− 10.40 ± 9.98	**0.005**	0.15
LDL (mg/dl)						
Pre-intervention	111.74 ± 22.29	114.63 ± 20.22	110.81 ± 28.22	110.87 ± 20.53	0.944	–
Post-intervention	109.48 ± 22.77	97.74 ± 18.9	94.86 ± 23.78	92.93 ± 15.65	0.057	0.378
P-value^▀^	0.155	** < 0.001**	** < 0.001**	** < 0.001**	–	–
Change▓	− 2.26 ± 6.83	− 16.89 ± 11.68^b^	− 15.96 ± 14.82^a^	− 17.93 ± 10.06^a^	** < 0.001**	**0.035**
HDL (mg/dl)						
Pre-intervention	48.25 ± 5.58	47 ± 6.39	47.14 ± 6.77	46 ± 6.37	0.734	–
Post-intervention	50.15 ± 5.34	56.47 ± 6.14^b^	54.9 ± 6.8^a^	55.7 ± 6.3^a^	**0.007**	**0.016**
P-value^▀^	** < 0.001**	** < 0.001**	** < 0.001**	** < 0.001**	–	–
Change▓	1.90 ± 1.41	9.48 ± 4.14^c^	7.76 ± 6.40^c^	9.70 ± 3.23 ^c^	** < 0.001**	** < 0.001**
Non-HDL (mg/dl)						
Pre-intervention	138.25 ± 26.47	141.95 ± 24.91	135.38 ± 26.47	135.35 ± 21.81	0.834	–
Post-intervention	134.1 ± 26.47	119.24 ± 24.91	117.76 ± 31.79	115.25 ± 21.81	**0.036**	0.34
P-value^▀^	**0.009**	** < 0.001**	** < 0.001**	** < 0.001**	–	–
Change▓	− 4.15 ± 6.42	− 22.71 ± 13.27^c^	− 17.62 ± 15.14^a^	− 20.10 ± 10.27^a^	** < 0.001**	**0.003**
HDL to LDL ratio						
Pre-intervention	0.45 ± 0.13	0.42 ± 0.11	0.47 ± 0.21	0.43 ± 0.11	0.712	0.192
Post-intervention	0.48 ± 0.14	0.6 ± 0.14	0.63 ± 0.23	0.62 ± 0.15	**0.025**	0.132
P-value^▀^	** < 0.001**	** < 0.001**	** < 0.001**	** < 0.001**	**–**	**–**
Change▓	− 0.03 ± 0.04	− 0.18 ± 0.1^c^	− 0.16 ± 0.15^b^	− 0.19 ± 0.08^c^	** < 0.001**	**0.002**

Data were presented as mean ± standard deviation

*HDL* high‐density lipoprotein-cholesterol, *LDL* low-density lipoprotein, *Non-HDL* non-high‐density lipoprotein

▓The changes (pre-post intervention) calculated based on the difference of variables from the baseline (pre- intervention) to the end (post intervention) of the study

P < 0.05 was considered significant difference

^▀^P-value was calculated for the comparison variables within group using paired t-test

P-value was calculated for the comparison variables between four groups using one-way analysis of variance (one-way ANOVA) with post hoc (LSD) analysis

^*^Adjusted P-value was calculated using ANCOVA; Adjusted for PA levels at the baseline, changes in BMI and weight

Significant changes with placebo group indicated by ^a^adjusted p < 0.05, ^b^adjusted p ≤ 0.01, ^c^adjusted p ≤ 0.001

As set out in Table 3 (within-group comparisons), curcumin, zinc, and “curcumin and zinc” groups revealed a significant improvement for post-intervention serum lipid profiles.

The placebo group showed a notable difference in post intervention TG (p = 0.001), HDL, HDL/LDL ratio (p ≤ 0.001), and non-HDL (p = 0.009) without any significant difference in TC (p = 0.155) and LDL (p = 0.103) levels (Table 3).

There was no significant difference in pre-intervention lipid profiles between the four groups.

After removing cofounders’ effects (baseline PA and changes in BMI and weight), a remarkable difference was found only for post intervention HDL (adjusted p = 0.016) between groups, without any significant difference for post-intervention TG (adjusted p = 0.286), non-HDL (adjusted p = 0.34), LDL (adjusted p = 0.378), TC (adjusted p = 0.583), and HDL/ LDL ratio (adjusted p = 0.132) between groups (Table 3).

Serum HDL levels significantly increased in the curcumin (adjusted p = 0.002), zinc (adjusted p = 0.016), and “curcumin and zinc” (adjusted p = 0.015) groups compared to the placebo group after adjusting cofounders’ effects (Additional file 1: Table A3).

After removing the confounders’ effect (baseline PA and changes in BMI and weight), the changes of all lipid profiles (pre-post) including serum TG (adjusted p = 0.001), LDL (adjusted p = 0.035), HDL (adjusted p ≤ 0.001), non-HDL (adjusted p = 0.003), and HDL/LDL ratio (adjusted p = 0.002) except serum TC (adjusted p = 0.15) showed a significant difference between four groups. In comparison to the placebo group, three supplement groups sustained a remarkable improvement for LDL, HDL, non-HD, and HDL/LDL ratio after removing the cofounders’ effects. The p-values were depicted in Additional file 1: Table A3. After removing the cofounders’ effects, changes (pre-post) in TG were revealed a significant difference only in the curcumin group in comparison to the placebo group (adjusted p = 0.001).

## Discussion

The main results of the present 90-days trial indicated that there was a significant difference between the groups for (1) the changes in BMI, weight, TG, LDL, HDL, non-HDL, and HDL/LDL ratio and (2) post-intervention HDL. Given that no significant difference for changes in ALT and AST and any reports of severe adverse reactions during the trial, the safety of the prescribed combination was represented.

Despite lifestyle recommendations, hypocaloric diet, and ongoing patient follow-up, the mean decrease in energy in each group was less than 500–1000 kcal (the values of recommended) and the changes in PA and dietary intake were not significant between groups, but the change (pre-post intervention) in weight and BMI showed a shift towards their improvement. Therefore, it may emanate from the positive effects of supplements on weight and BMI. It is notable that the undesirable impacts of quarantine on lowering PA cannot be ignored due to the prohibition of leaving home and the closure of gym, fitness and sports club, and park and the restrictions on social activity [26, 27].

It seems under the supervision of a nutritionist along with the closure of bars, restaurants, and coffee shops, remote staff, and enough time to prepare foods in the home due to COVID-19 quarantine may have led to the healthy selection of foods (better quality) without the remarkable changes in the amount of food (quantify) which contributed to the improvement of some of serum lipid profiles in the placebo groups.

After adjusting cofounder effects, the post-intervention comparisons of the serum lipid profiles showed there was a significant difference only for serum HDL between groups. This finding can present the important role of cofounders as well as the supplements on serum lipid levels during the intervention.

The progression of prediabetes to T2DM emanates from oncoming disturbed glucose and lipid metabolism [5]. On the other hand, the previous studies illustrated a high prevalence of dyslipidemia in prediabetes and T2DM [3, 4]. However, a correlation was reported between dyslipidemia prevalence with age, sex, education level, smoking status, alcohol drinking status, and obesity [4], the mean of the aforementioned variables was similar among all participants in the present trial. Therefore, the present findings may be related to the effect of the supplements.

As previously published [11], curcumin and zinc co-supplementation significantly improved some glycemic parameters such as IR and IS. Therefore, in addition to their indirect effects on lipid profiles via the improvement of IR and IS, other mechanisms may be illustrated the positive effects of the supplements on the lipid profile.

Given that the control cofounder in the present trial, it can be stated that the studied supplementations were presented the main role in improving the serum lipid profiles.

In agreement with the present results, a meta-analysis (2017) showed curcumin and turmeric decrease the risk factors of CVD through lowering TG and LDL in prediabetes, metabolic syndrome, T2DM, and hypertension [10]. Moreover, a trial conducted by Panahi et al. [8], touched upon an improvement in the changes of serum HDL, TC, and non-HDL and but no significant difference in TG and LDL changes after 1000 mg-curcumin intake in patients with T2DM. In another trial carried out by Thota et al. [28], the curcumin alone (1000 mg) or curcumin (1000 mg) with omega-3 did not alter serum lipid profiles in the patients with IFG and IGT.

In some previous investigations, an improvement in some serum lipid levels (TC, HDL, LDL, and TG) was illustrated following zinc supplementation [15, 17, 18] in different populations. Furthermore, the findings of a meta-analysis conducted by Asbaghi et al. [17] presented the beneficial impacts of zinc supplementation on HDL in both studies duration less or more than 12-weeks. Also, zinc supplementation decreased serum TG, TC, and LDL only in the studies with 12 weeks or less. It is notable that they pointed out the highest impact of zinc supplementation was on a dosage of less than 100 mg [17]. While the present trial did not show the beneficial effects of zinc supplement alone on serum TC and LDL and changes of TG in comparison to the placebo group, zinc supplementation with curcumin improved all serum lipid profiles except serum TC levels compared to the placebo group.

The conflicting findings of zinc or curcumin effects on serum lipid profiles can emanate from bioavailability and dose of the supplement, study duration, variety in the studied population (race, age, sex, history of diseases and etc.), several methodological limitations, the usage of supplement alone or with other nutrients (co-supplementation).

Some mechanisms that can justify the effect of curcumin or zinc supplements are separately described: curcumin can reduce serum TG through the different mechanisms, including increasing the gene expression of adiponectin and peroxisome proliferator-activated receptor alpha (PPAR-α), peroxisome proliferator-activated receptor gamma (PPAR-γ), cholesteryl ester transfers protein, and lipoprotein lipase activity. Curcumin can also reduce serum LDL by suppressing the LDL receptor gene expression through enhancement in PPAR-γ activation. In addition, curcumin reduces TC by affecting enzyme pathways of cholesterol metabolism [29].

Zinc may play effective roles in serum lipid profiles through several molecular mechanisms. The proposed mechanisms of directly and indirectly zinc action are as follows: (1) zinc can affect the activity of pancreatic β-cells through transporter expression; hence, regulating insulin storage and secretion; (2) zinc can improve insulin sensitivity or insulin resistance through (a) increasing the phosphorylation of insulin-receptor substrates at the adipocytes [12], (b) lipolysis inhibition in adipose tissues, which lead to a decrease in fatty acids released and finally regulate lipoprotein synthesis (VLDL and LDL secretion) from the liver; and (3) zinc can affect the gene expression of enzymes involved in hepatic lipid homeostasis leading to regulate lipid synthesis and utilization in mitochondria and peroxisomes [30].

The main limitation of the present trial was the participant recruitment was from single-center and the single dosage of each supplement was used. Further, a part of the research was done during quarantine conditions (COVID-19), the quarantine may have a desirable and undesirable impact on the present trial. The present study is unique in its novelty due to the use of zinc and curcumin co-supplements on serum lipid profiles of prediabetic patients.

It was suggested to conduct further RCTs with regard to the recruitment of patients with a longer history of prediabetes due to the appearance of macro and microvascular complications of prediabetes may need to longer time (the duration mean (CI) of prediabetes diagnosis in this trial was 2.32 [2.02, 2.62] months). Moreover, the serum lipid profiles of participants at the baseline were in the normal range. The different results may find in recruiting the participants with an abnormality in serum lipid profiles at the baseline.

## Conclusion

The present trial was reported the beneficial effects of curcumin and zinc co-supplementation on serum HDL and the changes in serum TG, LDL, HDL, non-HDL, HDL/LDL ratio, BMI, and weight among patients with diabetes. Therefore, it may play a potential role in the prevention of macro and microvascular complications.

It is suggested that multicenter and long-term clinical trials be conducted to confirm the serum lipid-improving properties of curcumin and zinc co-supplements in prediabetic patients with dyslipidemia. Moreover, it recommended examining whether co-administration of curcumin and zinc in other doses and forms can be useful for the prevention of diabetic macro and microvascular complications.

## Supplementary Information

**Additional file 1:** **Table A1.** The biochemical measurements of the participants at the baseline. **Table A2.** Comparison of changes in liver enzymes, anthropometry measurements, physical activity, and dietary intake between the groups. **Table A3.** Results of the pairwise comparisons of BMI, weight, and serum lipid profiles for significant ANCOVA.

## Data Availability

The datasets used and/or analyzed during the current study are available from the corresponding author on reasonable request.

## Abbreviations

ADA: American Diabetes Association
ALT: Alanine Transaminase
ANCOVA: Analysis of Covariance
AST: Aspartate Aminotransferase
BMI: Body Mass Index
CONSORT: Consolidated Standards for Reporting Trials
DBP: Diastolic Blood Pressure
ELISA: Enzyme-Linked Immunosorbent Assay
FPG: Fasting Plasma Glucose
IPAQ-SF: Short form of International Physical Activity Questionnaire
IQR: Interquartile range
IRCT: Iranian Registry of Clinical Trials
IR: Insulin resistance
IS: Insulin Sensitivity
LDL-C: Low-density lipoprotein cholesterol
MET: Metabolic Equivalent of Task
Non-HDL: Non-high density lipoprotein-cholesterol
One-way ANOVA: One-way Analysis of variance
PA: Physical activity
PPAR-α: Peroxisome proliferator-activated receptor alpha
PPAR-γ: Peroxisome proliferator-activated receptor gamma
RCT: Randomized Clinical Trial
SBP: Systolic Blood Pressure
SD: Standard Deviation
SPSS: Statistical Software Package
T2DM: Type 2 Diabetes Mellitus
