# Supplementary material for: Curcumin and zinc co-supplementation along with a loss-weight diet can improve lipid profiles in subjects with prediabetes: a multi-arm, parallel-group, randomized, double-blind placebo-controlled phase 2 clinical trial
Source: Diabetol Metab Syndr. 2022 Jan 28;14:22. doi: 10.1186/s13098-022-00792-2 (PMC8796182; doi:10.1186/s13098-022-00792-2)
Supplement: Supplementary file 1 — Additional file 1: Table A1. The biochemical measurements of the participants at the baseline. Table A2. Comparison of changes in liver enzymes, anthropometry measurements, physical activity, and dietary intake between the groups. Table A3. Results of the pairwise comparisons of BMI, weight, and serum lipid profiles for significant ANCOVA. [file 13098_2022_792_MOESM1_ESM.doc]

**Table S.1.**  The biochemical measurements of the participants at the baseline

| **Variables** | **Groups** | | | | **P** |
| --- | --- | --- | --- | --- | --- |
|  | **Placebo**  **N= 20** | **Curcumin**  **N= 21** | **Zinc**  **N= 21** | **Curcumin & Zinc**  **N= 20** |  |
| **Fasting plasma Glucose** (mg/dl) | 112 ± 11.48 | 108.81± 12.36 | 108.67 ± 11.88 | 112.23 ± 9.47 | 0.6 |
| **HbA1C (%)** | 5.9 ± 0.23 | 5.87 ± 0.29 | 6 ± 0.31 | 6 ± 0.34 | 0.35 |
| **Alanine transaminase** (IU/L) | 25.29 ± 5.91 | 25.76 ± 6.41 | 24.62 ± 5.38 | 25.43 ± 4.49 | 0.93 |
| **Aspartate transaminase** (IU/L) | 23.77 ± 4.97 | 24.90 ± 5.73 | 23.05 ± 4.94 | 22.14 ± 5.60 | 0.39 |
| **Triglyceride** (mg/dl) | 132.55 ± 25.96 | 131.48 ± 26.16 | 125.76 ± 25.74 | 126.1 ± 27.64 | 0.776 |
| **Cholesterol** (mg/dl) | 186.5 ± 24.91 | 188.95 ± 23.5 | 182.52 ± 26.11 | 181.35 ± 19.98 | 0.716 |
| **LDL** (mg/dl) | 111.74 ± 22.29 | 114.63 ± 20.22 | 110.81 ± 28.22 | 110.87 ± 20.53 | 0.944 |
| **HDL** (mg/dl) | 48.25 ± 5.58 | 47 ± 6.39 | 47.14 ± 6.77 | 46 ± 6.37 | 0.734 |
| **Non-HDL** (mg/dl) | 138.25 ± 26.47 | 141.95 ± 24.91 | 135.38 ± 26.47 | 135.35 ± 21.81 | 0.834 |
| **HDL to LDL ratio** | 0.45 ± 0.13 | 0.42 ± 0.11 | 0.47 ± 0.21 | 0.43 ± 0.11 | 0.712 |

HDL: high‐density lipoprotein-cholesterol; LDL: low-density lipoprotein; Non-HDL: non-high‐density lipoprotein.

Data are presented as mean ± standard deviation (SD) for quantitative variables and frequency (%) for qualitative variables.

P-value was calculated for the comparison variables between four groups using one-way analysis of variance (one-way ANOVA).

^#^ P-value was calculated for the comparison variables between four groups using Kruskal-Wallis.

P-value < 0.05 was considered significant.

**Table S.2.**  Comparison of changes in liver enzymes, anthropometry measurements, physical activity, and dietary intake between the groups.

| **Variables** | **Groups** | | | | **p** | **Adjusted P*** |
| --- | --- | --- | --- | --- | --- | --- |
|  | **Placebo**  **N= 20** | **Curcumin**  **N= 21** | **Zinc**  **N= 21** | **Zinc & curcumin**  **N= 20** |  |  |
| **Daily Energy** (kcal/day) | -252.50 ± 174.95 | -269.29 ± 105.84 | -261.19 ± 183.14 | -285.25 ± 124.69 | 0.915 | 0.94 |
| **Carbohydrate** (g) | -42.25 ± 29.36 | -39.28 ± 26.75 | -28.81 ± 21.96 | -46.50 ± 29.61 | 0.195 | 0.218 |
| **Fat** (g) | -7.55 ± 8.82 | -11.41 ± 8.68 | -10.31 ± 9.14 | -6.72 ± 7.31 | 0.253 | 0.151 |
| **Protein** (g) | -3.89 ± 18.52 | -2.37 ± 19.47 | -13.29 ± 20.16 | -9.68 ± 16.23 | 0.212 | 0.157 |

Data were presented as mean ± standard deviation.

P was calculated for the comparison variables between four groups using one-way analysis of variance (one-way ANOVA) with post hoc (LSD) analysis.

*Adjusted P-value was calculated using ANCOVA or nonparametric ANCOVA; Adjusted for physical activity levels at the baseline.

**Table S.3.** Results of the pairwise comparisons* of BMI, weight, and serum lipid profiles for significant ANCOVA

| **Variables** | **Groups** | | | | | |
| --- | --- | --- | --- | --- | --- | --- |
|  | **Curcumin** | | **Zinc** | | **Zinc & curcumin** | |
|  | **P** | **adjusted P♦** | **P** | **adjusted P♦** | **P** | **adjusted P♦** |
| **Triglyceride** (mg/dl) (Post-intervention) | 0.003 | NS | 0.101 | NS | 0.031 | NS |
| **Total. Cholesterol** (mg/dl) (Post-intervention) | NS | NS | NS | NS | NS | NS |
| **LDL** (mg/dl) (Post-intervention) | NS | NS | NS | NS | NS | NS |
| **HDL** (mg/dl) (Post-intervention) | 0.002 | 0.002 | 0.016 | 0. 016 | 0.006 | 0.015 |
| **Non-HDL** (mg/dl) (Post-intervention) | 0.034 | NS | 0.02 | NS | 0.008 | NS |
| **HDL to LDL ratio** (Post-intervention) | 0.027 | NS | 0.007 | NS | 0.011 | NS |
| **Change of Triglyceride** (mg/dl) | 0.002 | 0.001 | NS | NS | NS | NS |
| **Change of Cholesterol** (mg/dl) | 0.01 | NS | 0.016 | NS | 0.011 | NS |
| **Change of LDL** (mg/dl) | < 0.001 | 0.009 | < 0.001 | 0.019 | < 0.001 | 0.015 |
| **Change of HDL** (mg/dl) | < 0.001 | < 0.001 | < 0.001 | < 0.001 | < 0.001 | < 0.001 |
| **Change of Non-HDL** (mg/dl) | < 0.001 | < 0.001 | < 0.001 | 0.03 | < 0.001 | 0.023 |
| **Change of HDL to LDL ratio** | < 0.001 | 0.001 | < 0.001 | 0.006 | < 0.001 | 0.001 |
| **Change weight** (Kg) | 0.01 | 0.025◙ | 0.01 | 0.011◙ | < 0.001 | < 0.001◙ |
| **Change BMI** (Kg/m^2^) | 0.003 | 0.007◙ | 0.008 | 0.011◙ | 0.001 | 0.001◙ |

ANCOVA: Analysis of covariance; BMI: Body mass index; HDL-C: high‐density lipoprotein-cholesterol; LDL-C: low-density lipoprotein cholesterol; NS: Non significant; Non-HDL: non-high‐density lipoprotein-cholesterol; VLDL: Very low-density lipoprotein cholesterol.

*Each group compared to the placebo group.

NS: p ≥ 0.05;

P < 0.05 was considered significant difference.

**♦**Adjusted for physical activity levels at the baseline, changes in BMI and weight.

◙Adjusted for physical activity levels at the baseline.

P-vales were calculated by ANCOVA with post hoc (LSD) analysis.
